# Preliminary Evidence of Blood DNA Methylation Changes in Pregnant Women Adhering to a Mediterranean Diet

**DOI:** 10.3390/epigenomes10010012

**Published:** 2026-02-13

**Authors:** Grace Tavelli, Nikki Schultz, Joanna Brisbane, Nina Kresoje, Samantha Lodge, Jeremy K. Nicholson, Nicola J. Armstrong, Desiree Silva, Nina D’Vaz, David Martino

**Affiliations:** 1 The Kids Research Institute Australia, Perth Children’s Hospital, Nedlands, WA 6009, Australia; grace.tavelli@student.uts.edu.au (G.T.); nikki.schultz@thekids.org.au (N.S.); joanna.brisbane@thekids.org.au (J.B.); nina.kresoje@thekids.org.au (N.K.); desiree.silva@thekids.org.au (D.S.);; 2 Joondalup Health Campus, Joondalup, WA 6027, Australia; 3 School of Medical and Health Sciences, Edith Cowan University, Joondalup, WA 6027, Australia; 4 Centre for Computational and Systems Medicine, Health Futures Institute, Murdoch University, Perth, WA 6150, Australia; sam.lodge@murdoch.edu.au (S.L.); j.nicholson@imperial.ac.uk (J.K.N.); 5 Institute of Global Health Innovation, Imperial College London, London SW7 2AZ, UK; 6 School of Population Health, Curtin University, Perth, WA 6102, Australia; 7 School of Biomedical Sciences, The University of Western Australia, Crawley, WA 6009, Australia

**Keywords:** Mediterranean diet, pregnancy, epigenetics, methylation

## Abstract

Background/Objectives: Consumption of a Mediterranean diet (MD) has been associated with reduced incidence of non-communicable diseases and reduced overall mortality, with epigenomic effects representing plausible mediators. The aim of this pilot study was to explore potential epigenetic associations between DNA methylation markers in blood and adherence to an MD in pregnancy. Methods: Fifty-two pregnant women with high or low adherence to an MD throughout pregnancy, who participated in the BioMood ORIGINS study, were selected using an extremes-of-exposure design. DNA methylation (DNAm) profiles from whole blood were generated using the TWIST human methylome panel. We conducted both genome-wide and candidate gene-based differential methylation analyses to identify epigenetic variations between the study groups. Furthermore, we explored potential associations between blood methylation patterns and circulating inflammatory markers (GlycA, GlycB and SPC) previously observed to exhibit differential abundance in the same cohort of women. Results: There were no genome-wide significant differences in methylated dinucleotides between MD groups (*p*-value < 5 × 10^−8^); however, a region-based analysis identified 2210 differentially methylated regions (DMRs) (FDR < 0.05, absolute maximum logFC > 1) annotated to 1537 genes, significantly enriched in metabolic, inflammatory and neuronal signaling pathways. Leveraging publicly available data, we replicated nine novel DMR associations. Changes in circulating phospholipid inflammatory markers were significantly associated with a small methylation difference in Lipin-1 (*LPIN1)*, albeit with a small effect size (*p*-value < 5 × 10^−8^). A look-up analysis of previously reported MD-associated genes in this cohort detected small but statistically significantly different methylation of CpGs located within collagen type XVIII alpha 1 (*COL18A1)* and peroxisome proliferator-activated receptor gamma, coactivator 1 beta (*PPARGC1B)* gene regions. Conclusions: We provide preliminary evidence for modest methylation changes in specific genes associated with adherence to an MD.

## 1. Introduction

The Mediterranean diet (MD), characterized by a high intake of fruits, vegetables, whole grains, legumes, nuts, and olive oil and moderate consumption of fish and dairy products, has received attention for its health-promoting effects. Epidemiological studies consistently demonstrate that adherence to the MD is associated with a significantly reduced incidence of chronic non-communicable diseases (NCDs), such as cardiovascular disease, type 2 diabetes, and various cancers, and a lower all-cause mortality [1,2,3,4,5]. These protective effects are largely attributed to the MD diet’s anti-inflammatory properties, the ability to promote favorable gut microbiota, and the potential to modulate metabolic pathways [6]. Promoting adherence to an MD during pregnancy may be a promising prenatal strategy to support healthy pregnancies and for NCD risk reduction in both the mother and the offspring. Inflammation in pregnancy can predispose one to an elevated risk of pregnancy complications and NCDs in later life, and therefore reducing inflammation through dietary strategies could have beneficial effects on both the mother and offspring. While specific mechanisms are still unclear, epigenetic changes may mediate the MD diet’s effects on the host immune system.

Previous studies have shown that dietary metabolites typically associated with an MD can induce epigenetic changes in immune cells in proportion to the intake of various bioactive nutrients [7]. Flavonols, a class of phytochemicals, are found in high amounts in onions, broccoli and tomatoes and other fruits and vegetables commonly consumed in the MD and have been shown to influence miRNA expression patterns related to inflammation [8,9]. Sulforaphane (an organosulfur compound found in cruciferous vegetables) decreases DNA methylation levels in the nuclear factor erythroid 2-related factor 2 (*NRF2*) promoter and has been shown to increase the antioxidant and anti-inflammatory capacity in neurodegenerative diseases [10,11]. Lignans, found in high quantities in extra-virgin olive oil, have been shown to influence epigenetic regulation, including DNA demethylation, and may indirectly affect histone modifications such as H3K9me3, which are associated with heterochromatin formation and chemo-preventive effects [12]. Adherence to an MD in adults participating in the PREDIMED-Navarra randomized controlled trial was associated with differential methylation of CpG base modifications in the *COL18A1* gene encoding for the alpha chain of type XVIII collagen, and *PPARGC1B* transcription factor stimulant was implicated in type 2 diabetes [13]. These diet-induced epigenetic modifications can impact the function and responsiveness of immune cells at the nuclear level, thereby influencing systemic inflammation.

BioMood is an observational study exploring how consumption of an MD during pregnancy influences the gut microbiome, metabolome and inflammation. Previous work by our group on this cohort has demonstrated that adherence to an MD during pregnancy is characterized by shifts in amino acids and increased concentration of gut-microbial metabolites and is inversely associated with biomarkers of systemic inflammation, GlycA and GlycB [14]. The study identified distinct differences in the inflammatory marker supramolecular phospholipid composite (SPC). SPC_1_ is derived from phospholipids contained within the cardioprotective HDL-4 (density = 1.125–1.210 kg/L) subfraction, which were modified in the High-Mediterranean-Diet-Adherence (HMDA) group compared to the Low-Mediterranean-Diet-Adherence (LMDA) group [14]. SPC_2_ is derived from phospholipid contained within HDL1-3 (density = 1.063–1.125 kg/L) and SPC_3_ is derived from phospholipid contained within LDL. The present study builds upon this work to explore whether genome-wide blood DNA methylation changes were detectable in women with high versus low adherence to an MD throughout pregnancy. We conducted both hypothesis-free and hypothesis-driven analyses, examining whether serum metabolite changes observed in this cohort were associated with epigenetic changes in circulating immune cells. We focused on the *COL18A1* and *PPARGC1B* genes for our candidate gene analysis due to their biological relevance in pregnancy and potential interactions with the Mediterranean diet. *COL18A1* is involved in angiogenesis and extracellular matrix organization, processes critical for healthy pregnancy outcomes. *PPARGC1B* plays a role in energy metabolism and mitochondrial function, which are influenced by dietary patterns. Previous research has linked these genes to dietary patterns and pregnancy outcomes, providing a strong foundation for our analysis [13]. Our findings provide preliminary evidence for blood epigenetic changes that warrant further study.

## 2. Results

### 2.1. Description of the Cohort

The demographic characteristics of those included in the study are depicted in Table 1. The ethnic profile of the cohort was mixed, consisting of mostly Caucasian and South Asian ancestries. All women were taking common pregnancy supplements for iron, folate and vitamin D. Inflammatory morbidities (allergies, asthma, inflammatory bowel diseases) were generally low (less than 10% of the population) and equally distributed between groups.

### 2.2. Sequencing Quality Control and Exploratory Analysis

**Figure 1:**
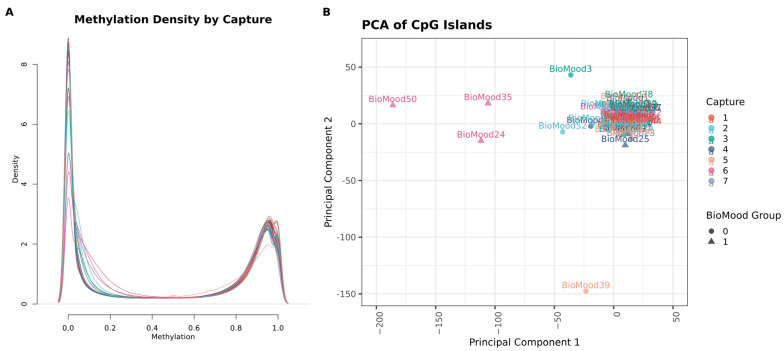
Diagnostic plots of sample quality control. (**A**) Density plot illustrating the distribution of genome-wide methylation levels across all samples. The x-axis represents methylation ratios expressed as beta values, ranging from unmethylated (0) to fully methylated (1). The y-axis indicates the density of these ratios. Each sample is color-coded according to the microarray slide (capture pool) it was processed on, with eight samples per slide. (**B**) Principal component analysis (PCA) of methylation levels across CpG island genomic regions. Each point represents an individual sample, plotted using the first two principal components. Samples are color-coded by capture pool and grouped according to MDA status. Four samples—BioMood35, BioMood24, BioMood50, and BioMood39—were identified as outliers.

The whole-blood DNA methylation sequencing depth averaged +196 million reads per sample. An average of 89.49% base calls per sample were of a quality score greater than or equal to Q30 (99.9% base call accuracy). During quality control analysis, four samples within the same target-capture pool were identified as poor quality based on visual inspection of Beta density plots and multidimensional scaling (MDS) analysis (Figure 1). These were removed from the data set along with CpGs with zero variance, retaining 48 samples, with 7.59 million CpG measures for analysis. We derived principal components to understand major sources of variation in this data set and correlated the top five components with clinical measures of subject age, BMI and MDA group (Supplementary Figure S3). Participant age was significantly correlated with component 2 (r = −0.34, *p* = 0.02) and MDA group had weak evidence of anti-correlation with component 5 (r = 0.25, *p* = 0.08). Regression models were thus adjusted for the first four components as covariates.

### 2.3. Epigenome-Wide Association Analysis of MDA Diet Group

**Figure 2:**
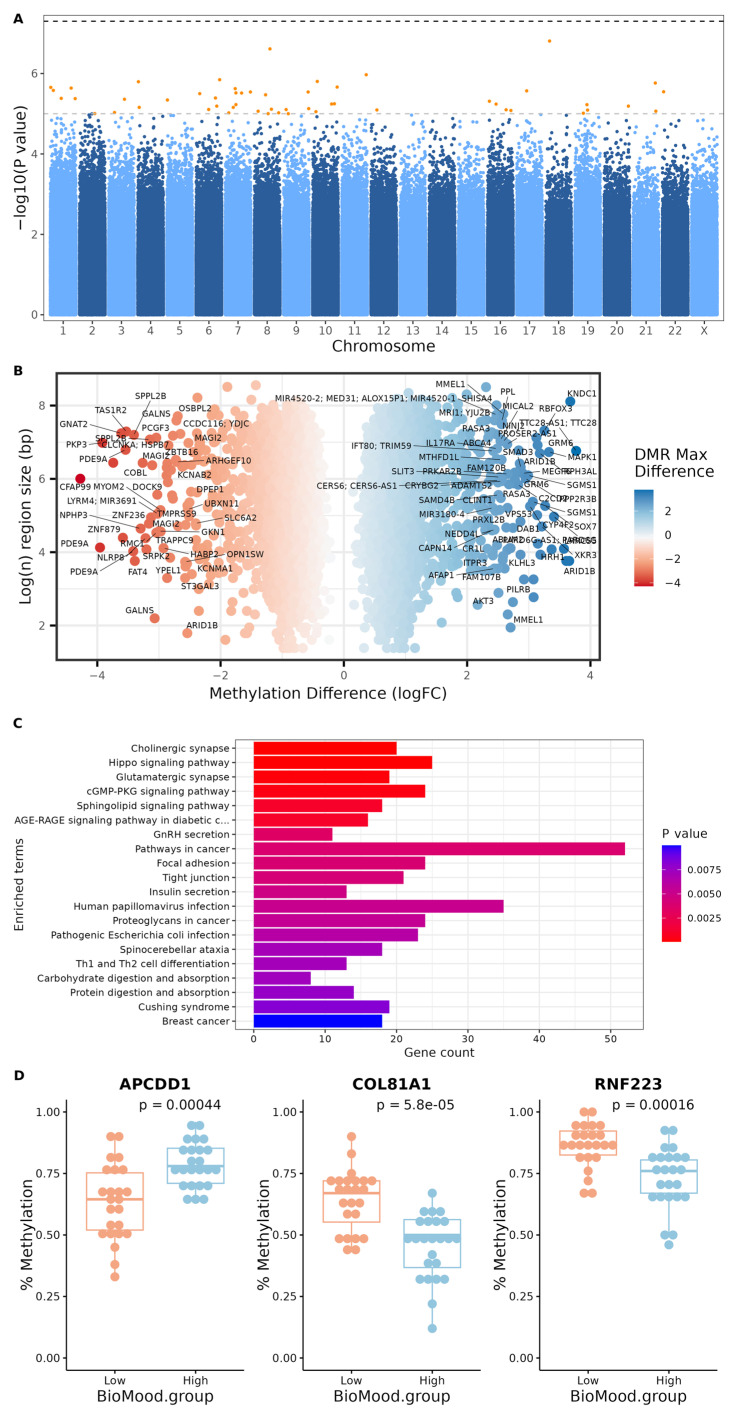
Integrative analysis of differential DNA methylation in BioMood groups. (**A**) Manhattan plot of differentially methylated CpG sites: the vertical axis represents the log 10 (unadjusted *p*-values) from the logistic regression analysis, while the horizontal axis indicates the chromosomal location of each CpG site. Horizontal dashed lines represent thresholds for suggestive (light grey) and significant associations (black); suggestive associations are colored orange. (**B**) Volcano plot of methylation differences: distribution of differentially methylated regions (DMRs) with an FDR *p* < 0.05 and maximum absolute logFC > 1 by log region size and logFC methylation difference. Points are colored by maximum differences between groups (HMDA vs LMDA), with blue dots showing an increase in methylation in the HMDA group, and red dots showing decreased methylation. Labels highlight key gene loci. (**C**) Pathway enrichment analysis: bar plot showing enriched pathways associated with differentially methylated genes with an FDR *p* < 0.05 and maximum absolute logFC > 1. The x-axis indicates gene count, and bars are colored by *p*-value significance. (**D**) Boxplots of top differentially methylated CpG sites: percent methylation levels for three lead CpG sites (*APCDD1*, *COL81A1*, and *RNF223*) comparing high and low Mediterranean Diet Adherence. *p*-values (Wilcoxon) for each site are indicated above the respective plots. HMDA = High-Mediterranean-Diet-Adherence group, LMDA = Low-Mediterranean-Diet-Adherence group; logFC = log fold change.

We applied a genome-wide binary logistic regression analysis comparing MDA groups to initially search for associations across all chromosomes. No individual CpGs reached the threshold for genome-wide significance (*p*-value < 5 × 10^−8^); however, 53 CpG sites met the threshold for a suggestive association (*p*-value < 1 × 10^−5^) (Figure 2A), albeit the effect sizes were very small (Table 2, Supplementary Table S1). Of the 53 differentially methylated CpGs, 12 were annotated to promoter regions, and 13 were within CpG islands. A region-based analysis identified 2210 differentially methylated regions (DMRs; FDR < 0.05 and maximum logFC > 1) (Figure 2B, Supplementary Table S2). Gene set enrichment analysis on the significant DMRs identified over-representation of pathways related to lipid metabolism (glycosphingolipid biosynthesis, hormone-sensitive lipase-mediated triacylglycerol hydrolysis, cAMP signaling), immune function (Th1 and Th2 cell differentiation, platelet activation, AGE-RAGE signaling), and neurodevelopmental processes (cholinergic synapse, synaptic vesicle cycle, axon guidance) (Figure 2C).

### 2.4. Replication Analysis of Novel MD-Associated DMRs

To explore the generalizability of the novel DMRs associated with MD adherence we leveraged publicly available data from Apron et al. [13], who examined changes in peripheral blood DNA methylation over a 5-year Mediterranean diet intervention in 36 older adults (mean age ~67 years) at high cardiovascular risk from the Spanish PREDIMED trial—a population differing substantially from our cohort of healthy pregnant women. We aligned this analysis by restricting PREDIMED Illumina HumanMethylation450 k array data to 5865 CpGs that overlapped with our DMRs based on genomic coordinates. We then fitted a binary logistic model, comparing the combined Mediterranean diet intervention groups (*n* = 48) to the low-fat diet control group (*n* = 24), adjusting for the first four principal components. Of the 13 DMRs, 9 showed evidence of replication (FDR < 0.05 and consistent direction of effect, Table 3).

### 2.5. Look-Up Analysis with Prior Mediterranean Diet Methylation Studies

**Figure 3:**
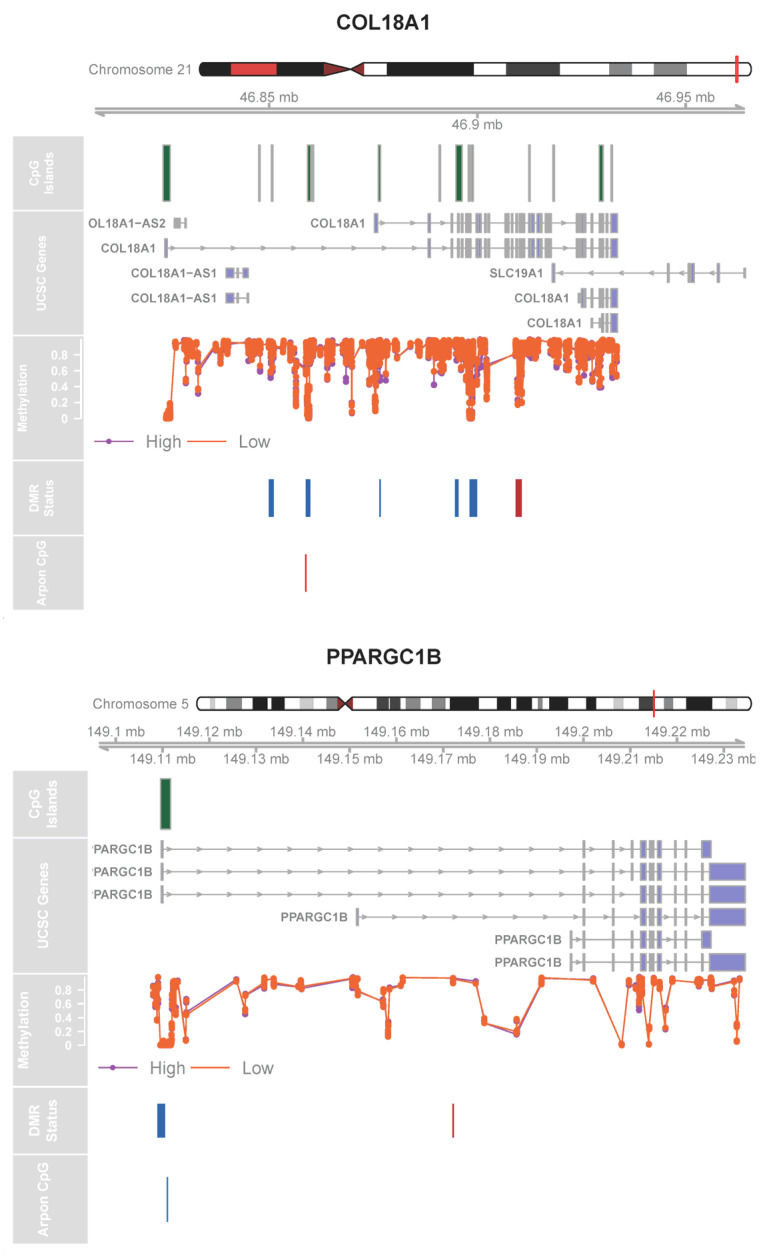
Genomic tracks of differentially methylated regions (DMRs) in *COL18A1* and *PPARGC1B* genes previously associated with Mediterranean diet adherence. Genomic tracks displaying DNA methylation patterns at the *COL18A1* (chromosome 21) and *PPARGC1B* (chromosome 5) loci. Top tracks show gene structure and CpG island annotations. Middle tracks display the DMR identified in the current study, with methylation differences between High- and Low-Mediterranean-Diet-Adherence groups indicated by color (red = hypermethylation in high-adherence group; blue = hypomethylation in high-adherence group). Bottom tracks show the location of CpGs previously reported as differentially methylated in association with a Mediterranean diet by Arpón et al., with the direction of effect indicated by color (red = hypermethylation; blue = hypomethylation).

We also conducted a look-up analysis focusing on CpGs in our cohort mapping to genes previously identified by Arpón et al. [13] as associated with MD adherence. We found moderate evidence (*p* value ≤ 1 *×* 10^−5^) of differential methylation at two genes, *COL18A1* and *PPARGC1B*, in CpG-level analysis and significant DMRs (FDR < 0.05 and maximum logFC > 1) in genes reported by Arpón et al., including *COL18A1* and *PPARGC1B* (Figure 3, Supplementary Table S2).

### 2.6. Association Between Inflammatory Markers and Methylation Levels

#### 2.6.1. Glycoprotein Analysis

We also conducted hypothesis-driven analysis, employing linear regression to examine whether the circulating inflammatory markers—GlycA, GlycB, SPC_1_, SPC_2_ and SPC_3_—that were previously shown to vary between the LMDA and HMDA groups [14] were associated with any changes in site-specific DNA methylation. To address potential co-linearity, we performed a correlation analysis of GlycA and GlycB, which showed they were highly positively correlated (r = 0.9549, *p*-value < 0.01, Supplementary Figure S3) and therefore we used GlycA as the independent variable. Genome-wide linear regression models revealed 83 CpG sites with suggestive associations (*p*-value < 1 × 10^−5^); however, none survived stringent multiple testing adjustment (Supplementary Table S3). No significant DMRs were identified in region-based analysis.

#### 2.6.2. SPC Lipoprotein Analysis

**Figure 4:**
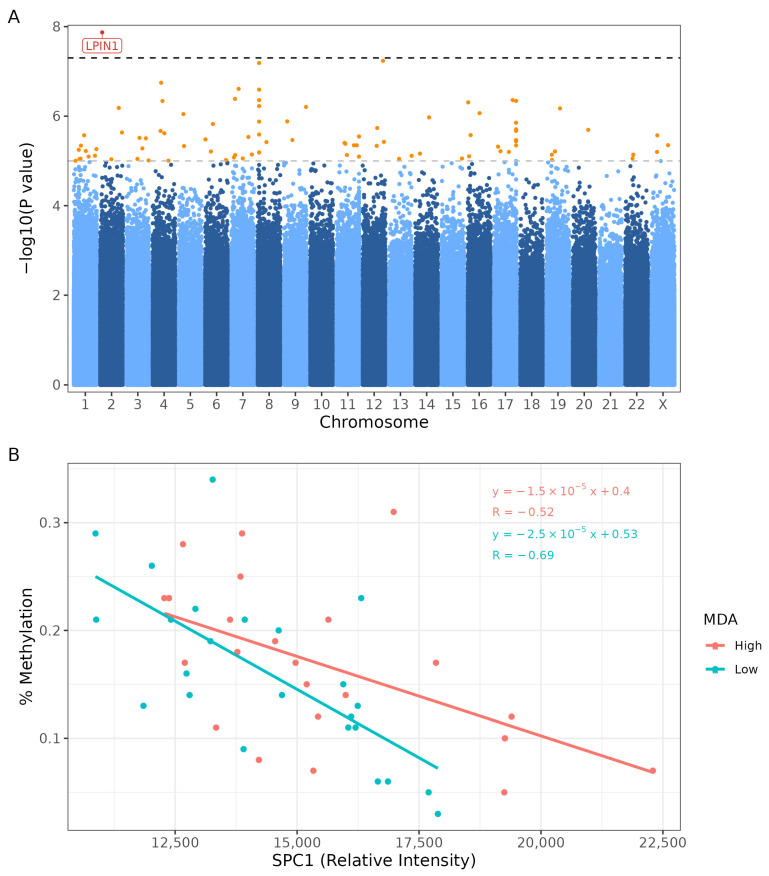
Differentially methylated CpG sites associated with SPC_1_ levels. Genome-wide linear regression detected one significantly different methylated CpG site within gene *LPIN1*, with strong evidence for association with circulating SPC_1_ metabolite levels (*p*-value < 5 × 10^−8^). (**A**) Manhattan plot of differentially methylated CpG sites; orange = suggestive association (*p* < 1 × 10^−5^)^,^ red = genome-wide significance (*p* < 5 × 10^−8^). (**B**) Scatterplot of relative SPC_1_ concentration against % methylation of LPIN1, with lines representing linear regression fit for each MDA group (HMDA in orange, LMDA in blue), demonstrating an inverse relationship (HMDA r = −0.52, LMDA r = −0.69). HMDA = High Mediterranean Diet Adherence; LMDA = Low Mediterranean Diet Adherence.

A Pearson correlation analysis between SPCs identified that SPC_2_ and SPC_3_ levels are highly positively correlated (r = 0.8459, *p*-value < 0.01, Supplementary Figure S3), and SPC_1_ was independent of SPC_2_ and SPC_3_. Therefore, subsequent analysis focused on SPC_1_ and SPC_3_. Genome-wide linear regression detected one significantly differentially methylated CpG site within gene Lipin-1 (*LPIN1*) with strong evidence for association with circulating SPC_1_ levels (*p*-value < 5 × 10^−8^, Figure 4). A further 90 CpG sites exhibited associations with changes in SPC_1_ levels (Supplementary Table S4). Our analysis of SPC_3_ showed no genome-wide significant associations and 102 CpG modifications with suggestive associations (Supplemental Table S5). Consistent with the GlycA regional analysis, we found no significant DMRs for either SPC_1_ or SPC_3._

## 3. Discussion

### 3.1. Summary of Key Findings

This pilot study explored the relationships between Mediterranean diet adherence in pregnancy and whole-blood DNA methylation using a multi-faceted approach. Despite the inherent limitations of a small sample size, robust signals were identified in specific areas with a priori biological plausibility. We identified robust signals in biologically plausible pathways and generated novel hypotheses for larger-scale studies.

### 3.2. Hypothesis-Free Analysis Findings

No site-specific associations reached genome-wide significance, though 53 CpG sites met suggestive thresholds with small effect sizes. This is consistent with subtle effects typically observed in nutritional epigenetics, particularly when using mixed cell tissues such as whole blood. Future studies will require substantially larger sample sizes for robust CpG-level associations at the genome scale.

Differentially methylated region analysis proved a more powerful approach, identifying 2210 DMRs representing coordinated methylation changes across genomic loci. Although absolute effect sizes were modest, nine DMRs showed concordant signals with the PREDIMED trial despite substantial differences in cohort profiles and methylation platforms, providing tentative evidence for reproducibility.

Gene set enrichment analysis revealed over-representation of pathways with clear mechanistic relevance to the observed metabolomic phenotypes. Pathways related to lipid metabolism (glycosphingolipid biosynthesis, hormone-sensitive lipase-mediated triacylglycerol hydrolysis, cAMP signaling) align with the shifts in circulating phospholipid inflammatory markers (SPC) observed in this cohort. Similarly, enrichment of immune-related pathways (Th1 and Th2 cell differentiation, AGE-RAGE signaling, platelet activation) is consistent with the anti-inflammatory effects of Mediterranean diet adherence reflected in altered GlycA levels. Notably, several neurodevelopmental pathways (cholinergic synapse, axon guidance, synaptic vesicle cycle) were also enriched, which may reflect maternal metabolic adaptations during pregnancy or circulating signals of fetal origin—an intriguing finding warranting further investigation.

Several individual DMR-associated genes support these pathway-level findings. *TAB1* encodes a key adaptor protein in the TAK1 signaling complex, mediating NF-κB activation and pro-inflammatory cytokine production. *TMEM184A* encodes a heparin receptor implicated in anti-inflammatory signaling in the vascular endothelium. *PDE9A* and *PDE1C* regulate the cGMP-PKG pathway, with inhibition of both associated with improved cardiovascular outcomes through modulation of calcium homeostasis. *AKT3* and *KCNN2* participate in glucose metabolism and insulin secretion, while *PILRB* regulates innate immune responses. Collectively, the convergence of replicated DMRs on inflammatory, cardiovascular, and metabolic pathways provides preliminary support for epigenetic mechanisms underlying the health benefits of Mediterranean diet adherence.

### 3.3. Hypothesis-Driven Analysis Findings

Consistent with previously published findings [13], we observed moderate evidence for differential methylation within *COL18A1* and *PPARGC1B*, genes involved in metabolism, adipogenesis and inflammation [15]. Effects sizes were small and inconsistent in direction, likely due to the mixed cell composition of whole blood.

We also investigated associations with circulating inflammatory lipoprotein markers that were previously shown to be modified by Mediterranean diet adherence in this cohort [14]. SPC_1_ levels—an NMR-derived measure of lipoprotein-bound phospholipids linked to cardiovascular risk—were associated with methylation changes at *LPIN1*. This gene encodes lipin-1, a phosphatidate phosphatase that regulates the balance between triglyceride storage and phospholipid biosynthesis. Methylation at this locus suggests a plausible candidate for functional follow-up and a potential molecular link between dietary exposure, epigenetic regulation, and the altered phospholipid profiles observed metabolically.

### 3.4. Strengths and Limitations

A novel aspect of this study was the use of methylation sequencing, which offers inherently more quantitative data and substantially broader coverage of the mammalian genome compared to microarray platforms. Our cohort was robustly selected based on prior evidence of metabolic changes associated with Mediterranean diet adherence, enriching for biological signal. Notably, nine DMRs were replicated in the independent PREDIMED trial despite substantial differences in cohort characteristics and methylation platform, suggesting these signals are not spurious findings driven by the limitations of a small pilot study.

The primary limitations are the small sample size and profiling of mixed cell tissue, increasing the likelihood of false negatives and attenuating effect sizes. We lacked functional data such as gene expression to support preliminary findings, as viable mRNA was not obtainable from snap-frozen whole blood. The extremes-of-exposure design may limit generalizability, and replication in larger, more diverse cohorts is required. The cross-sectional design precludes causal inference, and we could not disentangle the effects of highly correlated phospholipid and glycoprotein species. Further investigation with longitudinal sampling and mediation analyses will be needed to establish directionality and causality.

### 3.5. Conclusion

This study provides valuable hypotheses for future exploration of the epigenetic mechanisms associated with diet in pregnancy and suggests potential links between circulating inflammatory markers and blood methylation. Longitudinal studies with larger sample sizes using purified cell populations would be needed to replicate associations and establish biological significance. Compelling preliminary findings around SPC_1_ associations and candidate genes *COL18A1*, *IFI30* and *PPARGC1B* provide a foundation to guide future research.

## 4. Materials and Methods

### 4.1. Study Cohort

Participants included in the BioMood study were nested within the ORIGINS parent cohort, a longitudinal study of family health outcomes, commencing in pregnancy [16,17]. The present study utilized available whole-blood samples collected at the 36th week of pregnancy from eligible mothers. The BioMood study entry criteria have been previously published [14]. The pregnant women who were recruited to the BioMood study comprised women who had completed a modified 13-item Mediterranean Diet Questionnaire (MDQ) twice during pregnancy, with scores that varied by no more than 2 points. A total of 52 women met eligibility criteria, which included 25 control participants within the Low-Mediterranean-Diet-Adherence (LMDA) group (an MDQ score equal to or less than 4 at 2 timepoints) and 27 case participants within the High-Mediterranean-Diet-Adherence (HMDA) group (an MDQ score equal to or greater than 8 at 2 timepoints). Participants were excluded if they did not have available biological samples for analysis, were exposed to antibiotics at any time during pregnancy, reported use of anti-depressant or anti-anxiety medication or had a pre-pregnancy BMI over 40 kg/m^2^. Study groups were balanced for potential confounding variables which include age, parity, education, work status, pre-pregnancy weight and pre-pregnancy BMI.

### 4.2. Serum Inflammatory Marker Analysis

Metabolic profiles were generated using 1H Nuclear Magnetic Resonance (NMR) spectroscopy. Further details on the acquisition, processing and analysis of metabolic profiles are described in the BIOMOOD study [14]. The NMR signal intensities of interest were glycoprotein signals GlycA and GlycB, and supramolecular phospholipid composite (SPC) peaks, which correspond to small HDL phospholipids (SPC_1_), larger HDL phospholipids (SPC_2_) and LDL phospholipid particles (SPC_3_).

### 4.3. DNA Extraction and Library Preparation

Genomic DNA was extracted from whole blood using either chemagic™ DNA Blood 400 Kit H96 (Revvity, Waltham, MA, USA; Cat. No. CMG-1091) on the chemagic™ 360 instrument (Revvity, Waltham, MA, USA) (*n* = 49), or the DNeasy Blood & Tissue Kit (QIAGEN, Hilden, Germany; Cat. No. 69504; *n* = 2), following manufacturers’ protocols (Supplementary Figure S1). DNA concentration and purity were measured using Qubit ™ dsDNA High-Sensitivity (HS) Assay Kit (Cat. No. Q33231, ThermoFisher Scientific, Waltham, MA, USA) on a Qubit fluorometer (ThermoFisher Scientific, Waltham, MA, USA). A total of 200 ng of genomic DNA per sample was fragmented to 275–325 bp using the Covaris E220 sonicator (Covaris, Woburn, MA, USA). Control DNA (0.5% methylated pUC19 and unmethylated lambda DNA) was included to assess enzymatic conversion efficiency.

Indexed pre-capture libraries were prepared using the NEBNext^®^ Enzymatic Methyl-seq Library Preparation Protocol (Twist Bioscience and NEB) following the manufacturer’s protocol. Eight libraries were pooled equimolarly for capture, balanced by diet group and BMI. Target enrichment was performed using the Twist Human Methylome Panel (Twist Bioscience, South San Francisco, CA, USA; Code: TWB105520), capturing 3.98 million CpG sites. Hybridization was carried out for 16 h at 60 °C, followed by washing and 6-cycle PCR amplification according to the Twist Targeted Methylation Sequencing Protocol. Library size and concentration were assessed using the Agilent 4200 TapeStation system with the D5000 ScreenTape Assay (Agilent Technologies, Santa Clara, CA, USA).

### 4.4. Sequencing

Final libraries were sequenced at the Genomics WA accredited facility on an Illumina NovaSeq 6000 (Illumina, San Diego, CA, USA) using the XP workflow on an S4 flowcell (2 × 150 (bp) reads) with a loading concentration of 253 pM and a 10%PhiX spike-in.

### 4.5. Bioinformatic Analysis: Pre-Processing

Raw methylation FASTQ files were processed using the nf-core/methylseq v2.6.0 pipeline. The methylseq pipeline was executed under Nextflow v23.10.0 [18] and the Human Genome Reference Consortium Human Build 37 (GRCh37) using the Bismark workflow (v0.24.0). Binary Alignment/Map (BAM) Index files were constructed using samtools version 1.13 [19]. The nf-core methylseq pipeline was run under the profile singularity and a custom configuration file adopted from the pre-configured Pawsey Setonix HPC settings, specifying the compute resources required for the job scheduler (slurm) [20] (Supplementary Figure S2).

### 4.6. Statistical Analysis

All statistical analyses were conducted in R language v4.4.1 and RStudio 2023.03.0+386 using base R functions (unless otherwise specified). Processing of Bismark methylation calls was performed using the bsseq package version 1.40.0 [21]. Bismark files were processed to filter any CpGs that were not represented in all samples and had a coverage of less than 5 or greater than 500 reads. Non-standard and off-target chromosome reads were removed from the set. Sample quality was assessed by examining genome-wide methylation patterns. Raw methylation data were extracted using the getMeth function from the minfi package (v1.50.0) and visualized with densityPlot [22]. Sex was predicted based on median methylation values of loci on chromosomes X and Y, calculated using chrSelectBSseq, getMeth, and colMedians, with females identified as those with less than 0.25 difference in median methylation between chromosomes.

Methylation data, stored as a bsseq object, were annotated to the Twist Human Methylome Panel Target BED File (Twist Bioscience, South San Francisco, CA, USA). Promoter regions, CpG islands, and enhancer regions were identified using build_annotations (annotatr package v1.30.0) and subsetByOverlaps [23]. For each region, methylation ratios were extracted using getMeth (bsseq package v1.40.0), and principal component analysis (PCA) was performed using prcomp. Samples identified as outliers in the PCA were excluded from further analysis (4 samples removed from the same capture pool). Methylation beta values were extracted using getMeth. M-values were calculated by log2-transforming the ratio of methylated to unmethylated read counts obtained via getCoverage. Batch effects were assessed by PCA on beta values. Associations between the top five principal components and clinical traits were evaluated using the WGCNA package (v1.72-5) [24]. Spearman correlations and corresponding *p*-values were calculated and visualized using labeledHeatmap. Pearson correlations between GlycA and GlycB, and among SPC_1_, SPC_2_, and SPC_3_, were assessed and visualized. Normality of metabolite distributions was also assessed using ggplots. SPC subregions were log-transformed prior to correlation analysis.

### 4.7. Hypothesis Testing

Genome-wide differential methylation analysis was performed using the limma package (v3.60.4) [25]. To identify CpG sites differentially methylated between low- and high-MDA groups, logistic regression models were fit to M-values and adjusting for the first four principal components. Empirical Bayes statistics were calculated, and top-ranked differentially methylated sites were identified. Effect sizes are reported on the Beta value scale. Differentially methylated CpG sites were deemed statistically significant at unadjusted *p* values < 5 × 10^−8^ and suggestive at unadjusted *p* values < 1× 10^−5^. Differentially methylated region (DMR) analysis was performed using the DMRcate package (v3.2.1) under default settings. DMRs were considered significant if they met a false discovery rate (FDR) threshold of less than 0.05 and had an absolute maximum methylation difference of logFC greater than 1. Gene set enrichment analysis was performed on genes annotated to significant DMRs using enrichR (v3.4) and the KEGG 2021 database.

An additional candidate gene analysis of CpG sites annotated to genes associated with MD published by Apron et al. [13] was conducted using logistic regression. Genes of interest included Eukaryotic Translation Elongation Factor 2 (*EEF2)*, RUNX Family Transcription Factor 3 (*RUNX3)*, Interleukin 4 Induced 1 (*IL4I1)*, mitogen-activated protein kinase (MAPK)-activated protein kinase 2 (*MAPKAP2)*, *COL18A1*, leptin receptor (*LEPR)*, pleiomorphic adenoma gene-like 1 (*PLAGL1)*, interferon-related developmental regulator 1 (*IFRD1)*, and *PPARGC1B.* Specific CpG sites annotated to these genomic coding regions based on genomic location (hg19 build) were extracted for analysis. The specific regions of interest and corresponding CpGs are available in the published codebase. Statistical significance was declared at a Bonferroni-corrected *p* value threshold of less than 0.05.

Linear regression models were used to test associations between genome-side CpG and metabolite levels (GlycA, GlycB, SPC_1_, SPC_2_, and SPC_3_), adjusting for MDA status and the first four principal components. As related metabolites exhibited co-linearity, models were further adjusted by incorporating correlated metabolites as adjustment variables. Differentially methylated sites were visualized using manhattan_plot from the ggmanh package (v1.8.0) [26].

## Figures and Tables

**Table 1:** Demographic information of participants in the BioMood study.

	Mediterranean Diet Adherence		
Variable	Low N = 25	High N = 27	*p*-value ^1^
Age in Years, Mean (SD)	31.3 (3.7)	32.8 (3.9)	0.154
Pre-pregnancy Weight, kg, Mean (SD)	74 (12)	71 (15)	0.346
Pre-pregnancy Body Mass Index (BMI), kg/m^2^, Mean (SD)	28.0 (4.2)	25.3 (5.4)	0.055
Parity, n (%)			0.601
0	14 (58%)	13 (52%)	
1	9 (38%)	9 (36%)	
2	1 (4.2%)	3 (12%)	
Education, n (%)			0.569
Bachelor	10 (40%)	12 (44%)	
Other	2 (8.0%)	2 (7.4%)	
Postgrad	6 (24%)	8 (30%)	
Trade	2 (8.0%)	4 (15%)	
Year 10	1 (4.0%)	0 (0%)	
Year 12	4 (16%)	1 (3.7%)	
Ethnicity, n (%)			0.244
Asian	0 (0%)	3 (11%)	
Australian	5 (20%)	3 (11%)	
European	19 (76%)	20 (74%)	
New Zealander	0 (0%)	1 (3.7%)	
North American	1 (4.0%)	0 (0%)	
Pregnancy Morbidity, n (%)	10 (67%)	7 (47%)	0.461

^1^ *p*-values: *t*-test for continuous variables; Chi-square test for categorical variables. BMI missing for three participants: two in low and one in high diet groups.

**Table 2:** Top ten differentially methylated CpG sites associated with MDA.

Chr	CpG Location	Effect Size	*p* Value	Nearest Gene (bp)
18	10,453,700	−0.088	1.55 × 10^−7^	APCDD1 (−924)
8	99,318,378	−0.048	2.44 × 10^−7^	KCNS2 (−120,871), NIPAL2 (−11,758)
11	129,488,337	−0.184	1.07 × 10^−6^	TMEM45B (−197,376), BARX2 (+242,503)
6	167,504,840	−0.047	1.42 × 10^−6^	CCR6 (−31,416), FGFR1OP (+92,171)
10	22,048,252	−0.050	1.57 × 10^−6^	MLLT10 (+224,981), DNAJC1 (+244,401)
4	719,927	−0.040	1.60 × 10^−6^	PCGF3 (+20,374), CPLX1 (+100,058)
21	46,924,305	−0.194	1.72 × 10^−6^	SLC19A1 (+38,079), COL18A1 (+48,903)
10	134,829,071	0.048	2.17 × 10^−6^	TTC40 (−72,983), GPR123 (−72,337)
1	1,011,561	−0.145	2.21 × 10^−6^	RNF223 (−1875)
1	201,749,312	−0.115	2.30 × 10^−6^	IPO9 (−48,957)

Note: Effect size = logFC of the methylation values from HMDA–LMDA; i.e., positive effect size means there is higher methylation associated with HMDA. Chr = chromosome. Nearest gene association rule: basal + extension (5 kb upstream and 1 kb downstream, up to 1 kb mx extension; hg19 genome.) HMDA = High-Mediterranean-Diet-Adherence group, LMDA = Low-Mediterranean-Diet-Adherence group; bp= base pair.

**Table 3:** Replicated differentially methylated regions (DMRs) showing consistent direction of effect between the current study and Arpón et al.

Chr	Genomic Position (hg19)	Width (bp)	Gene	No. CpGs	Mean Diff (Current) (logFC)	Mean Diff (Arpón) ΔBeta	FDR (Current)	FDR (Arpón)
1	6,149,031–6,149,591	561	*KCNAB2*	42	−0.311	−0.012	3.19 × 10^−5^	7.10 × 10^−4^
2	20,868,568–20,872,603	4036	*GDF7*	358	−0.857	−0.119	1.62 × 10^−93^	3.83 × 10^−14^
6	28,058,067–28,059,776	1710	*ZSCAN12P1*	89	−0.407	−0.078	1.13 × 10^−15^	1.24 × 10^−7^
7	1,587,483–1,589,887	2405	*TMEM184A*	149	0.112	0.046	2.28 × 10^−6^	1.55 × 10^−3^
16	33,964,398–33,966,445	2048	*LINC00273*	385	0.181	0.036	4.13 × 10^−3^	6.80 × 10^−3^
20	43,378,440–43,379,595	1156	*KCNK15*	144	0.309	0.071	8.66 × 10^−4^	2.08 × 10^−10^
20	43,936,663–43,937,468	806	*MATN4*; *RBPJL*	50	−0.480	−0.028	1.85 × 10^−5^	3.59 × 10^−2^
20	58,507,904–58,509,611	1708	*SYCP2*; *FAM217B*	331	0.137	0.028	1.34 × 10^−10^	3.58 × 10^−2^
22	39,784,481–39,785,135	655	*TAB1*	97	−0.550	−0.050	1.46 × 10^−5^	3.41 × 10^−2^

## Data Availability

Analysis scripts have been made publicly available on github: https://github.com/ClinicalEpigenetics/BioMood_DNAm (accessed on 4 February 2026). The methylation data are available on request from the ORIGINS cohort (contact the corresponding author) due to restrictions on public deposition of DNA methylation data to accord with participant consent.

## Supplementary Materials

The following supporting information can be downloaded at: https://www.mdpi.com/article/10.3390/epigenomes10010012/s1, Figure S1: Flow Chart of Methylation Sequencing Experiment; Figure S2: Flow Chart of Bioinformatic Analysis; Figure S3: Pearson Correlation Analysis of Metabolites; Figure S4: Heatmap of Principal Components vs Phenotypes; Table S1: Suggestive significant differentially methylated CpGs for HMDA vs LMDA; Table S2: Differentially methylation regions for HMDA vs LMDA; Table S3: Suggestive differentially methylated CpGs for GlycA; Table S4: Suggestive and significant differentially methylated CpGs for SPC1; Table S5: Suggestive differentially methylated CpGs for SPC3.

## Abbreviations

The following abbreviations are used in this manuscript:

BMI	Body mass index
DMR	Differentially Methylated Region
DNAm	DNA methylation
FDR	False Discovery Rate
logFC	Logarithmic Fold Change
MD	Mediterranean diet
MDQ	Mediterranean Diet Questionnaire
(L/H)MDA	(Low/High) Mediterranean Diet Adherence
NCD	Non-communicable disease
NMR	Nuclear Magnetic Resonance
*NRF2*	Nuclear factor erythroid 2-related factor 2
PCA	Principal component analysis
SPC	Supramolecular phospholipid composite
